# PD-1/PD-L1信号通路在非小细胞肺癌免疫逃逸及其治疗中的研究进展

**DOI:** 10.3779/j.issn.1009-3419.2014.10.05

**Published:** 2014-10-20

**Affiliations:** 350025 福州，南京军区福州总医院肿瘤科 Nanjing Military Command Fuzhou General Hospital, Fuzhou 350025, China

**Keywords:** 肺肿瘤, 程序性死亡分子1, 程序性死亡分子1配体, 免疫治疗, 调定点阻滞剂, 免疫逃逸, Lung neoplasms, Programmed death 1, Programmed death 1 ligand, Immuno-therapy, Checkpoint inhibitors, Immune escape

## Abstract

非小细胞肺癌（non-small cell lung cancer, NSCLC）是肿瘤相关性死亡率第一的恶性肿瘤。虽然近年来靶向治疗进展迅速，但很多基因未突变的患者不能从中受益。目前免疫治疗已成为肿瘤治疗的新方向，它能通过刺激机体免疫系统提高抗肿瘤免疫效应。研究显示免疫检查点分子：程序性死亡分子1（programmed death 1, PD-1）、程序性死亡分子1配体（PD-1 ligand, PD-L1），与肿瘤发生、发展密切相关，在NSCLC中有重要的临床意义。PD-1/PD-L1信号通路的激活有助于肿瘤免疫逃逸，而阻断该通路可以增强机体内源性抗肿瘤免疫效应。目前越来越多的临床试验显示免疫检查点阻滞剂抗PD-1、抗PD-L1抗体在治疗NSCLC中的良好疗效性和安全性。本综述旨在回顾及总结近年来PD-1/PD-L1信号通路及其阻滞剂在NSCLC中的研究进展。

## Abstract (en)

Lung cancer is the leading cause of cancer-related mortality worldwide. Despiting the great progress on target agents, majority of people who do not harbor a mutation could not get benefit from them. Immunotherapy, through stimulating the body's immune system to improve the antitumor immunity effect, has been a new therapeutic method for non-small cell lung cancer (NSCLC). Study had been reported that immune checkpoint molecules, including programmed death-1 (PD-1)/PD-ligand (L) 1 axis, are closedly related with cancer generation and development, and play a key role on clinical significance of NSCLC. Activation of PD-1/PD-L1 pathway contributes to tumor immune escape, and block PD-1/PD-L1 pathway can enhance endogenous antimuor immunity. Currently increasing clinical trials suggested that immune checkpoint inhibitors, including anti-PD-1 and anti-PD-L1 monoclonal antibodies turned out to be beneficial and safe in NSCLC. Here, we provide a review on the progress of PD-1/PD-L1 pathway and immune checkpoint inhibitors in NSCLC.

肺癌是世界上最常见的恶性肿瘤之一。在我国，肺癌的发病率、死亡率均高居恶性肿瘤之首，分别为53.57/10万、45.57/10万，严重威胁着国民的健康^[1]^。非小细胞肺癌（non-small cell lung cancer, NSCLC）约占肺癌的84%，大部分患者就诊时已属晚期，其治疗手段比较局限，5年生存率仅为2%^[2, 3]^。近年来肿瘤免疫治疗研究突飞猛进^[4]^：机体免疫系统除了有免疫监视清除肿瘤细胞作用，在某些阶段还促进肿瘤免疫逃逸，在肿瘤发生、发展过程中扮演极其重要的角色。研究^[5]^显示程序性死亡分子1（programmed death 1, PD-1）/PD-1配体（PD-1 ligand, PD-L1）信号通路的激活可导致免疫抑制性肿瘤微环境形成，使肿瘤细胞逃避机体免疫监视和杀伤，而阻断PD-1/PD-L1信号通路可以逆转肿瘤免疫微环境，增强内源性抗肿瘤免疫效应。目前免疫检查点阻滞剂抗PD-1、抗PD-L1抗体因其特异性强、副作用低、肿瘤控制时间长等优点已成为治疗NSCLC新方向，在临床试验中不断取得突破性进展^[6-8]^。

## 1 免疫检查点分子与肿瘤

肿瘤的特性之一是能通过基因、表观遗传的改变导致一系列抗原的产生，人体免疫系统能够通过识别肿瘤特异性抗原、肿瘤相关抗原，产生T细胞免疫应答清除肿瘤细胞。T细胞的完全活化需要“双重信号”：首先，T细胞表面受体（T cell antigen receptor, TCR）识别由抗原提呈细胞（antigen-presenting cells, APCs）提呈的抗原肽-主要组织相容性复合体，使T细胞初步活化。进而，APC表面的共刺激分子与T细胞表面的相应共刺激分子结合，使T完全活化成为效应T细胞。在正常生理情况下，共刺激分子与共抑制分子之间的平衡，即免疫检查点分子的平衡，使T细胞的免疫效应保持适当的深度、广度，从而最大程度减少对于周围正常组织的损伤，维持对自身组织的耐受、避免自身免疫反应。然而，肿瘤细胞可异常上调共抑制分子及其相关配体，例如PD-1、PD-L1，抑制T细胞的免疫活性，造成肿瘤免疫逃逸，导致肿瘤发生、发展^[9, 10]^。越来越多的证据^[6-8]^表明阻断共抑制分子与配体结合可以加强及维持内源性抗肿瘤效应，使肿瘤得到持久的控制。

目前临床研究最为透彻的免疫检查点分子有：细胞毒性T淋巴细胞相关抗原4（cytotoxic T lymphocyte-associated antigen 4, CTLA-4）、PD-1及PD-L1。而利用免疫检查点阻滞剂抗PD-1、抗PD-L1抗体阻断PD-1/PD-L1信号通路治疗晚期NSCLC在临床试验展现良好的疗效性、安全性。

## 2 PD-1/PD-L1信号通路及其生物学功能

PD-1是免疫球蛋白B7-CD28家族成员之一，由胞外段、疏水性跨膜区、胞内段组成，其胞内段含有免疫受体酪氨酸抑制基序（immunoreceptor tyrosine-based inhibitory motif, ITIM）、免疫受体酪氨酸转换基序（immunoreceptor tyrosine-based switch motif, ITSM）^[11]^。其中，ITSM的激活与效应性T细胞应答活性密切相关。PD-1可表达于活化的CD4^+^T细胞、CD8^+^T细胞、B细胞、自然杀伤T细胞、单核细胞和树突状细胞上^[10]^。另外，研究^[12]^显示PD-1也表达于调节性T细胞（regulatory T cell, Treg），并能促进Treg细胞的增殖，抑制免疫应答。

PD-1包括两个配体：PD-L1^[13]^、PD-L2（B7-CD, CD273）^[14]^。其中，PD-L1是PD-1的主要配体，在许多恶性肿瘤中高表达，包括NSCLC^[15, 16]^、黑色素瘤^[17]^、肾细胞癌^[18]^、前列腺癌^[19]^、乳腺癌^[20]^、胶质瘤^[21]^等。研究^[22, 23]^发现PD-L1在肿瘤浸润性树突状细胞、肿瘤浸润性淋巴细胞（tumour infiltrating lymphocytes, TILs）、肿瘤浸润性巨噬细胞中也表达。目前认为PD-L1的表达可能有两种机制^[5, 24]^：①由肿瘤的癌基因调控，也称为固有免疫抵抗，通过PI3K-AKT、EGFR、ALK/STAT3等信号通路诱导肿瘤细胞表达PD-L1，此时PD-L1的表达是持续性的，与肿瘤微环境中炎症反应无关^[25-27]^；②由肿瘤免疫微环境中的T细胞驱动的，亦称为适应性免疫抵抗，此时PD-L1是抗肿瘤免疫效应过程中产生的炎症信号诱发的，呈非持续性表达，可使机体免受感染引发的、免疫介导的组织损伤^[28]^。另外，最新的文献^[17]^报道显示，在黑色素瘤模型中，PD-L1的上调与CD8 T细胞密切相关，而并不依赖于癌基因信号。上述的研究或许能提示哪些患者更能从免疫检查点阻滞剂中受益，未来的研究需要阐明两种不同机制在不同肿瘤中的作用。值得注意的是，Butte等^[29]^的研究显示PD-L1除了结合PD-1，也可与激活的T细胞表面的CD-80（B7-1）结合，此时CD-80是作为受体而非配体传递负调节信号。相对于PD-L1，PD-L2仅在NSCLC、黑色素瘤、肾细胞癌等少数肿瘤细胞及浸润性免疫细胞中表达^[23]^，其具体免疫调节机制尚不完全明确，但PD-L1、PD-L2在信号通路中的功能或有重叠，值得未来进一步探究^[14]^。

研究^[13, 14, 30, 31]^表明，PD-L1作为B7家族中一个重要的T细胞共抑制分子，与PD-1结合后可通过ITSM募集酪氨酸磷酸酶SHP-2，进而去磷酸化TCR信号通路上的多个关键分子，抑制CD4 T、CD8 T细胞的增殖和活性，负性调节机体免疫应答过程。在健康的机体中，PD-1/PD-L1信号通路的激活可最大程度减少免疫反应对周围组织的损伤，避免发生自身免疫疾病^[10, 32]^。然而，PD-1/PD-L1信号通路激活可使肿瘤局部微环境T细胞免疫效应降低，从而介导肿瘤免疫逃逸，促进肿瘤生长^[5]^，其具体机制尚不完全清楚，可能有以下几个方面：①诱导T细胞耐受：PD-L1与PD-1持续作用可抑制TCR介导的停止信号，导致T细胞的外周耐受。利用抗PD-1、抗PD-L1抗体阻断两者相互作用，可降低T细胞的运动性、增加T细胞与树突状细胞作用时间，导致自身免疫性糖尿病形成^[33]^；②诱导T细胞凋亡：肿瘤细胞表达的PD-L1能通过与PD-1及其他受体结合增加抗原特异性T细胞的凋亡，阻断PD-L1能减少T细胞凋亡。另外，小鼠模型也显示PD-L1能促进活化的肿瘤效应性T细胞的凋亡^[34]^；③诱导T细胞耗竭：研究^[35]^显示：17.8%的肿瘤发生与慢性感染密切相关。而对慢性病毒感染的研究发现，PD-1在功能耗竭的T细胞上高表达，阻断PD-1/PD-L1信号通路可以恢复T细胞的增殖、分泌和杀伤功能^[36]^。最新的研究^[37]^表明：肿瘤微环境中的TILs的耗竭与肿瘤细胞、肿瘤来源的髓系细胞分泌的PD-L1有关，阻断PD-1/PD-L1信号通路可以增加效应性CD8 T细胞的功能，抑制Treg细胞和髓系来源抑制细胞的功能，加强抗肿瘤效应。因此，PD-1、PD-L1可能在T细胞耗竭中作用不可忽视；④增强Treg细胞的功能：PD-L1能通过下调mTOR、AKT、S6和ERK2的磷酸化及上调PTEN，促进诱发性Treg的产生、维持，从而抑制效应性T细胞活性^[12]^；⑤抑制T细胞增殖：PD-1可以通过选择性抑制RAS/MEK/ERK及PI3K/AKT信号通路，进而抑制细胞周期相关基因转录、蛋白表达，阻碍细胞周期进展及T细胞的增殖^[38]^；⑥可诱导性共刺激分子（inducible co-stimulatory molecule, ICOS）与PD-1的失衡：Sugita等^[39]^研究显示，生发中心树突状细胞、滤泡树突状细胞的功能发挥不仅仅依赖于T细胞的激活，也和PD-L1的负调节相关。ICOS、PD-1可同时共表达于某些活化的CD4 T细胞上，前者传递正刺激信号促进T细胞激活，而后者则相反，两者间的平衡决定T细胞的正常功能。因此，未来肿瘤免疫治疗的研究重点在于从根源上阻断PD-1/PD-L1信号通路的激活，维持正常T细胞功能，减少免疫逃避，从而提高抗肿瘤免疫效应。

近年来，众多基础试验也支持免疫检查点阻滞剂抗PD-1、抗PD-L1抗体在阻断PD-1/PD-L1信号通路的疗效。在小鼠模型中，利用抗PD-1抗体阻断PD-1/PD-L1信号通路可以降低促癌细胞因子的表达，增强效应性T细胞的功能，重塑肿瘤免疫微环境，使小鼠寿命延长^[26, 40]^。相关研究^[41, 42]^显示*PD-1*或*PD-L1*基因缺失的小模型不易发生自身免疫病，也进一步验证了阻断PD-1/PD-L1信号通路可以增强机体免疫应答。

## 3 PD-1、PD-L1在NSCLC中的临床意义

鉴于PD-1/PD-L1信号通路在肿瘤的免疫逃逸及其治疗中的重要意义，免疫检查点分子PD-1、PD-L1在NSCLC中的临床意义也得到广泛的研究。

回顾性分析^[22, 43]^显示在NSCLC中，肿瘤细胞过表达PD-L1提示侵袭性高、预后差，类似结论在肝癌、结直肠癌等肿瘤中均见报道^[44, 45]^。而Yang等^[46]^研究显示PD-L1过表达的Ⅰ期肺腺癌患者无复发生存时间更长；Velcheti等^[15]^研究提示PD-L1蛋白或RNA过表达的患者有更长的总生存时间（overall survival, OS），而与年龄、分期、组织类型无关。甚至有研究^[47]^认为PD-L1与NSCLC的预后无明显相关。各个研究的结果不一致可能与PD-L1的表达易受到检验试剂、检验方法、肿瘤样本质量、肿瘤类型及肿瘤异质性等因素影响有关，因此，目前PD-L1表达水平能否作为预后指标尚未得到一致肯定，有待研究进一步证实。

有研究表明PD-L1的表达可能与NSCLC免疫治疗的疗效相关。Ⅰ期临床试验^[6]^提示仅有少数NSCLC患者对于抗PD-1抗体（nivolumab）有效，PD-L1阳性、阴性患者的客观缓解率（objective response rate, ORR）分别为36%、0%，因此，PD-L1阳性被认为可能是潜在抗PD-1抗体治疗有效的生物学靶标。进一步的回顾性分析^[23]^提示：肿瘤细胞PD-L1的表达与浸润性免疫细胞明显相关，虽然与其他免疫抑制分子PD-1及PD-L2的表达亦相关，但它反映的是一个充满免疫活性的微环境，是抗PD-1抗体疗效评价最密切的独立因素。不过鉴于上述结果由回顾性分析得来且样本量较小，可靠性值得商榷。在最新的MPDL3280A和MK-3475的临床试验^[48, 49]^中也观察到，PD-L1高表达的NSCLC患者较低表达或阴性表达患者的ORR更高，但两者同样面临样本较小问题。Taube等^[28]^在黑色素瘤中也观察到PD-L1阳性较PD-L1阴性患者OS明显延长，提示PD-L1阳性的患者可能更受益。然而，临床试验^[48, 50, 51]^显示部分PD-L1阴性的NSCLC患者也能从抗PD-1、抗PD-L1治疗中受益，若将PD-L1阳性作为生物学靶标有可能剔除潜在免疫治疗获益人群。此外，PD-L1状态与ORR无明显相关也可见报道^[51]^。因此，PD-L1预测免疫治疗疗效有待于大样本Ⅱ期、Ⅲ期临床试验进一步验证。

近期报道^[52]^显示NSCLC患者服用靶向药物的疗效与PD-L1的表达密切相关，而与PD-1表达无关：*EGFR*基因突变且服用厄洛替尼或吉非替尼治疗的晚期NSCLC患者中，PD-L1阳性与PD-L1阴性对比，疾病进展时间（time to progression, TTP）明显延长（13.0个月*vs* 8.5个月，*P*=0.011），OS有延长趋势（29.5个月*vs* 21.0个月，*P*=0.752）。这项研究不仅有助于筛选靶向药物治疗的优势人群，而且也提示了未来研究抗PD-1、抗PD-L1抗体联合靶向药物治疗的潜在临床价值。

## 4 免疫检查点阻滞剂在NSCLC中的临床应用前景

### 4.1 抗PD-1抗体

#### 4.1.1 Nivolumab（MDX-1106, BMS-936558, ONO-4538）

Nivolumab是全人源化免疫球蛋白G4、抗PD-1抗体，它的出现改变了既往肺癌对于免疫治疗不敏感、疗效差的观念。2010年*J Clin Oncol*杂志报道了用MDX-1106阻断PD-1/PD-L1通路治疗难治性实体瘤的Ⅰ期临床试验^[53]^，研究共包括晚期转移性黑色素瘤、NSCLC、肾癌、去势抵抗前列腺癌和结肠癌等，结果显示MDX-1106可激活机体免疫效应，发挥有效的抗肿瘤作用，初步认为阻断PD-1/PD-L1通路是相对比较安全的。2012年新英格兰杂志发表的有关nivolumab的Ⅰ期临床试验（CA209-003, NCT00730639）^[6]^结果显示：在76例可评价的NSCLC患者中，所有剂量组的ORR为18%，疾病稳定时间大于24周的患者为7%。值得一提的是，55%的患者此前已接受了至少3次系统治疗。但是考虑到样本量少，须谨慎解读该数据。

Nivolumab在NSCLC的明显生存获益促使其Ⅰ期扩展性临床试验^[54]^的开展，该试验共入组129例此前接受过系统治疗的晚期NSCLC患者，试验分为1 mg/kg、3 mg/kg和10 mg/kg剂量组。结果显示：根据标准实体瘤的疗效评价标准（Response Evaluation Criteria in Solid Tumors, RECIST）1.0评估标准，所有剂量组的ORR为17%（22/129），中位OS为9.9个月，1年、2年生存率明显提高，高达42%、24%。在肿瘤初次评估（第8周）时即有50%（11/22）获得缓解，而且中位持续反应时间持久，高达74.0周（6.1周-133.9周）。进一步分析显示在所有患者中，3 mg/kg剂量组ORR最高，达24%，OS最长，为14.9个月，因此3 mg/kg被选为未来研究的标准剂量；而非鳞癌、鳞癌患者的OS无明显差异。药物相关的不良事件（adverse events, AEs）总发生率为41%，3/4级的严重药物AEs为5%，主要包括：皮肤（16%）、胃肠道反应（12%）、肺部（7%）。药物相关的肺炎发生率为（6%），3级/4级肺炎发生率为2%（2/129），试验早期有2例患者因肺炎死亡，因此，临床试验早期干预值得研究者重视。研究还提示：组织学类型、既往系统治疗次数 > 3次，年龄 > 70岁、*EGFR*、*Kras*基因状态并不影响Nivolumab的临床疗效。

Nivolumab的明显疗效引发了我们对于晚期NSCLC治疗的思考：免疫治疗作为一线还是二线使用？是单独应用还是与其他疗法联合应用疗效最佳？是否可作为转换维持治疗？两项相关的Ⅲ期临床试验NCT01673867、NCT01642004正在开展，分别对比nivolumab与多西他赛治疗鳞癌、非鳞癌疗效差异。另一项Ⅰ期临床试验NCT01454102正在招募中，研究nivolumab联合含铂类双药、厄洛替尼、贝伐珠单抗或抗CTLA-4抗体ipilimumab在不同类型NSCLC患者中的疗效。

#### 4.1.2 Lambrolizumab（MK-3475）

MK-3475也是人源化IgG4、抗PD-1抗体。Ⅰ期临床试验^[49]^纳入了38例至少接受过2次系统治疗的晚期NSCLC患者。给药方式为每3周给药1次，每次10 mg/kg，每9周行1次肿瘤影像检查，直到疾病发生进展为止。数据分析提示，第9周时大部分患者肿瘤即有缓解，根据RECIST 1.1评估标准，总ORR为21%，初步估计中位OS为51周，中位无进展生存期（progression free survival, PFS）为9.7周。亚组分析显示：鳞癌患者的ORR为33%，高于非鳞癌患者的16%，该结果是否提示鳞癌患者的免疫治疗疗效更佳，目前结论尚不清楚，有待于大样本临床试验验证。53%患者发生药物相关AEs，最常见的是皮疹（21%）、瘙痒（18%）和乏力（16%）。

目前MK-3475联合化疗或比对化疗运用于特定NSCLC人群的相关临床试验NCT01840579、NCT01905657也在积极开展中。

### 4.2 抗PD-L1抗体

#### 4.2.1 BMS-936559

BMS-936559是高亲和力、全人源化IgG4抗体，Ⅰ期临床试验NCT00729664^[7]^共纳入包括NSCLC、黑色素瘤、结直肠癌、肾细胞癌、宫颈癌、胰腺癌、胃癌、乳腺癌等207例晚期患者。结果显示只有NSCLC、黑色素瘤、肾细胞癌、宫颈癌获得持久肿瘤退缩，ORR为6%-17%；在75例晚期NSCLC患者中，有49例患者可评估，ORR为10%（5/49），24周PFS比率为31%。药物相关AEs多为1级-2级，包括皮疹、腹泻、输液反应、过敏反应、内分泌紊乱、眼干、肝功能异常等。

#### 4.2.2 MPDL3280A

MPDL3280A是人源化IgG4抗体，无抗体依赖的细胞介导的细胞毒作用（antibody-dependent cell-mediated cytotoxicity, ADCC），理论上可避免杀伤肿瘤直接激活的T细胞。Ⅰ期临床试验^[48]^结果显示：MPDL3280A在既往接受过系统治疗的晚期NSCLC中具有良好的耐受性及安全性，总ORR为23%（12/53），24周PFS比率为46%。此外研究提示MPDL3280A的疗效与EGFR状态、组织类型、先前接受治疗的次数无明显相关，而可能与PD-L1状态相关，PD-L1阳性与阴性患者ORR分别为80%（4/5）、14%（4/28）。相对于其他免疫检查点阻滞剂，MPDL3280A的AEs较轻，3级-4级AEs发生率为12%，包括疲乏、高血糖、缺氧，至今尚没有3级-5级肺炎、腹泻病例报告。

目前MPDL3280A相关两项的Ⅱ临床试验NCT01846416、NCT01903993正在招募中，期待进一步的结果报告指导晚期NSCLC的诊疗。

值得注意的是，Gajewski等^[55]^研究提示：免疫治疗或许应根据患者的不同肿瘤微环境特征，即炎症性或非炎症性T细胞亚型，而采取不同的干预模式。对于前者，肿瘤主要通过免疫系统抑制性通路逃避免疫杀伤，因此免疫检查点阻滞剂可能获得最佳疗效，而对于后者可能并不受益。此外，虽然抗PD-1、抗PD-L1抗体均作用于PD-1/PD-L1信号通路，但两者的作用靶点不同：抗PD-1抗体能阻断PD-1与PD-L1、PD-L2结合，却不能阻断PD-L1与CD80相互作用，抗PD-L1抗体能阻断PD-L1与PD-1、CD-80结合，却不能阻断PD-1与PD-L2的结合^[56]^；其次，两者的亲和力不同，导致对通路阻断程度、作用时间不同；最后，抗体亚型也不尽相同，例如IgG1抗体与IgG4抗体，两者介导的ADCC、补体依赖性细胞毒作用强度不同、对于T细胞的影响不同，这些都导致抗PD-1、抗PD-L1抗体疗效和毒性的差异^[57]^。因此，抗PD-1、抗PD-L1抗体的适用人群有待进一步研究，而两者的具体临床疗效差异有待于头对头临床试验证实。

### 4.3 联合免疫治疗

近年来，免疫检查点阻断剂联合治疗是否能获得最佳疗效成为研究热点。早期基础试验提示PD-1、CTLA-4联合阻断能增加肿瘤微环境TILs的表达，减少Treg细胞、髓系细胞的表达，促使抑制性肿瘤微环境的抗肿瘤免疫效应增强^[58]^。最新Ⅰ期临床试验^[59]^结果提示，相对于Nivolumab序贯抗CTLA-4抗体（Ipilimumab）方案组治疗晚期黑色素瘤，nivolumab、ipilimumab同时方案组的临床疗效更佳，后者的ORR可达40%，明显高于序贯组的20%。其次，同时方案组应用最大剂量伴可接受水平的不良事件时，53%患者获得客观缓解，所有患者肿瘤减小至少80%，效果明显。另外，还有研究^[60, 61]^显示抗PD-1或PD-L1抗体联合抗淋巴细胞激活基因3（lymphocyte activation gene 3, LAG3）抗体或IL-2治疗也能协同增强抗肿瘤免疫。但联合免疫治疗在NSCLC中的疗效有待临床试验结果进一步揭示。

## 5 小结

目前PD-1/PD-L1信号通路阻断在NSCLC中的重要作用得到广泛重视。免疫检查点阻滞剂迅速、持久、有效的药物反应、明显的生存获益具有里程碑式意义，为NSCLC的免疫治疗奠定了坚实的基础，也促进更多的基础及临床试验的扩展、深入。但同时也带给我们思考：PD-L1的预后和预测价值尚不明确，作为生物标记物还有待商榷，因此，探索肿瘤和机体免疫之间的关系，肿瘤微环境与疗效的关系，从而寻找稳定的生物标记物，筛选肿瘤免疫治疗优势人群，使治疗个体化、最优化，是未来亟需解决的问题；免疫治疗作为肿瘤治疗的新方向，已经成为NSCLC综合治疗的重要组成部分，其在不同阶段NSCLC的适应症及与其他免疫治疗联合的临床意义有待未来更多临床试验的广泛开展。
